# Thermodynamic analysis of a gamma type Stirling engine in an energy recovery system

**DOI:** 10.1016/j.enconman.2018.03.085

**Published:** 2018-06-01

**Authors:** Ayodeji Sowale, Athanasios J. Kolios, Beatriz Fidalgo, Tosin Somorin, Alison Parker, Leon Williams, Matt Collins, Ewan McAdam, Sean Tyrrel

**Affiliations:** School of Water, Energy and Environment, Cranfield University, Bedfordshire MK43 0AL, United Kingdom

**Keywords:** Energy recovery, Micro CHP, Nano Membrane Toilet, Stirling engine

## Abstract

The demand for better hygiene has increased the need for developing more effective sanitation systems and facilities for the safe disposal of human urine and faeces. Non-Sewered Sanitary systems are considered to be one of the promising alternative solutions to the existing flush toilet system. An example of these systems is the Nano Membrane Toilet (NMT) system being developed at Cranfield University, which targets the safe disposal of human waste while generating power and recovering water. The NMT will generate energy from the conversion of human waste with the use of a micro-combustor; the heat produced will power a Stirling engine connected to a linear alternator to generate electricity. This study presents a numerical investigation of the thermodynamic analysis and operational characteristics of a quasi steady state model of the gamma type Stirling engine integrated into a combustor in the back end of the NMT system. The effects of the working gas, at different temperatures, on the Stirling engine performance are also presented. The results show that with the heater temperature of 390 °C from the heat supply via conduction at 820 W from the flue gas, the Stirling engine generates a daily power output of 27 Wh/h at a frequency of 23.85 Hz.

## Highlights

• The performance of Stirling engine integrated to a micro-combustor in the NMT system was investigated.
• Energy recovery and power generation of 27 Wh/h from combustion of human faeces.
• The integrated position of the Stirling engine to the micro-combustor is highly paramount.
• Sensitivity of the performance of the Stirling engine to working gas temperature.
• Requirements for optimum performance of the Stirling engine for integration with micro-combustor.

## Nomenclature

${\text{A}}_{\text{d}}$: cross-sectional area of the piston (${\text{m}}^{2}$)
${\text{A}}_{\text{p}}$: cross-sectional area of the displacer (${\text{m}}^{2}$)
${\text{C}}_{\text{ap}}$: specific heat at constant pressure (J/kg K)
${\text{C}}_{\text{av}}$: specific heat at constant volume (J/kg K)
diss: heat loss due to frictional flow in the heat exchangers (W)
K: Kelvin
k: thermal conductivity (W/m K)
${\text{m}}_{\text{d}}$: mass of the displacer (kg)
m_p_: mass of the piston (kg)
${\text{T}}_{\text{k}}$: temperature of the cooler (K)
${\text{T}}_{\text{r}}$: temperature of the regenerator (K)
${\text{T}}_{\text{h}}$: temperature of the heater (K)
${\text{T}}_{\text{ck}}$: temperature of the compression space to cooler (K)
${\text{T}}_{\text{he}}$: temperature of the heater to expansion space (K)
${\text{T}}_{\text{rh}}$: temperature of the regenerator to heater (K)
${\text{T}}_{\text{kr}}$: temperature of the cooler to regenerator (K)
${\text{V}}_{\text{b}}$: bounce space volume (${\text{m}}^{3}$)
${\text{V}}_{\text{r}}$: volume of the regenerator $({\text{m}}^{3}$)
${\text{V}}_{\text{h}}$: volume of the heater (${\text{m}}^{3}$)
${\text{V}}_{\text{k}}$: volume of the cooler (${\text{m}}^{3}$)
${\text{V}}_{\text{e}}$: volume of the expansion space $({\text{m}}^{3}$)
${\text{V}}_{\text{c}}$: volume of the compression space $({\text{m}}^{3}$)
θ: crank angle (degree)
φ: phase angle (degree)
${\text{V}}_{\text{clc}}$: compression space clearance volume (${\text{m}}^{3}$)
${\text{V}}_{\text{cle}}$: expansion space clearance volume (${\text{m}}^{3}$)
$V_{\mathrm{sd}}$: piston swept volume $({\text{m}}^{3}$)
$V_{\mathrm{sd}}$: displacer swept volume $({\text{m}}^{3}$)
R: gas constant value
*d*: derivative
Q: heat transfer rate (W)
Q_heat_: heat transfer rate in heater (W)
Q_reg_: heat transfer rate in regenerator (W)
Q_cool_: heat transfer rate in cooler (W)
Q_cond_: heat transfer by conduction (W)
${\text{A}}_{\text{fs}}$: free surface area (${\text{m}}^{2}$)
P_e_: pressure in expansion space (MPa)
P_c_: pressure in compression space (MPa)
sht: shuttle loss (W)
*lir*: internal heat conduction loss (W)
$Q_{\mathrm{hdiss}}$: heat dissipation loss due to friction in the heater (W)
$Q_{\mathrm{rdiss}}$: heat dissipation loss due to friction in the regenerator (W)
$Q_{\mathrm{kdiss}}$: heat dissipation loss due to friction in the cooler (W)
*h*: heat transfer coefficient (W/m^2^ K)
Tq: torque (N m)

## 1 Introduction

Heat recovery and waste utilisation are rapidly advancing fields of research, due to the high priority currently given to energy generation and environmental sustainability. Waste heat from processing plants and the exhaust of engine systems is now typically recovered via an integrated energy system, and materials that would otherwise be considered as waste, are being explored for their energy potential. This is due to the increase in the demand for renewable energy production over the years as a result of increasing population and industrial development. Power generation (electricity) is the most feasible source of energy in the modern world and proves to be a drive for economic and human advancement. The recovery of energy from waste has been a primary target for power generation in recent years [1] and has paved the way for investigations into systems that can generate power from waste, especially for developing countries [2].

In the sanitation industry, the conventional flush toilet systems are undergoing a major design revision because of their extended infrastructure requirements such as sewer systems. Non-sewered technologies, often without or with limited necessity for flush water, are being investigated. Some of these novel toilet systems are described as functioning as thermochemical conversion units, where faeces are thermally treated to produce useful by-products [3]. It has been estimated that about 2.3 billion people in developing countries lack sufficient and suitable means of sanitation. A considerable reduction in mortality rate from outbreaks of infectious diseases in developed and developing countries could be prevented with improved and safer sanitation systems [4]. The Nano Membrane Toilet (NMT) is being developed to treat human waste into clean water and heat without the external supply of water, energy, and sewer. This unit requires the development of new technologies for power generation, and the use of human faecal material as an energy source is one example. The combined benefits of the novel systems embedded in this unit (i.e. membrane technology for urine filtration, and micro-combustor for continuous conversion of human faeces) can improve access to clean water and sanitation around the world, as well as enhance alternative and environmentally-friendly power generation for communities lacking basic amenities [5]. Therefore, heat recovery from combustion-based systems is important, and electrical energy is vital for the functioning of the self-sustaining/off-grid NMT system reliant on pumps and ignition systems.

Conversion of thermal energy to electrical energy in medium-scale commercial environments is mostly associated with gas engines, Rankine engines, microturbines, fuel cells and Stirling engines in a cogeneration system [6]. Heat recovery for power generation in household-scale applications can be accomplished with the use of an external combustion engine such as a Stirling engine that can function by using the heat generated from a gas stream at high temperature. Stirling engines have been considered for cogeneration systems due to certain features that give them greater advantage over other reciprocating engines, such as low vibration, very low emissions, high efficiency and the ability to utilise different forms of energy [7]. Stirling engines also operate in a closed, regenerative thermodynamic cycle [8] and they have been employed in various applications, such as combined heat and power (CHP) production, solar power generation, heat pumps, nuclear power for electricity generation, and geothermal energy [9]. The performance of the Stirling engine is based on its physical and geometrical features, the type and properties of the working gas, regenerator porosity and efficiency, dead volume, heat exchanger temperature, pressure drop, and heat and shuttle losses. The efficiency of the Stirling engine is usually between 30 and 40% based on operating temperature (working fluid temperature) from 686 °C to 800 °C and operating speed from 33 Hz to 67 Hz [10]. In the case of the NMT unit, the primary aim of the integration of the combustor with the Stirling engine is to utilise the excess heat from the former to power the engine, and convert to electricity by connecting to an alternator. The Stirling engine is considered over other options due to its compatibility with the micro-combustor of the NMT, high specific power and efficiency, and good performance at partial load; also due to features which are particularly advantageous for household applications such as simplicity, long-life cycle, low emission level, and low vibration and noise levels.

The disadvantages of Stirling engines are low compression ratio, working gas leakage and large volume. Certain approaches have been taken to increase the output power of the engines, such as the selection of the working gas, with the use of helium rather than hydrogen at high pressure, and increase in heat transfer surface area and internal heat transfer coefficient. Changes have also been made to the mechanical arrangements, such as the use of free piston Stirling rather than conventional Stirling engines; although the free piston Stirling engine has its own minimal disadvantages in connection with the stability of the mechanical elements, such as the damper and mechanical springs [11].

The application of a biomass energy conversion system using a Stirling engine is more flexible than the conventional biomass energy conversion with gas engines [12]. In addition, there have been recent developments on biofuel powered Stirling engines. The utilisation of bioenergy with the application of Stirling engines has proved to be a promising technology [13]. In Denmark, Carlsen and Bovin [14] developed and tested a 9 kW_e_ Stirling engine with wood gas as the fuel source and the engine generates about 10 kW and 24% of electric power and efficiency from 11 kW of shaft power. A wood chip fired boiler integrated with a 35 kW_e_ Stirling engine CHP system was run successfully in Austria [15]. A comparison of the use of the Stirling engine and organic Rankine cycle turbine for electricity generation from poultry waste was carried out by Cotana et al. [16] where the authors showed that the Stirling engine had a better capability of generating higher power output with the conversion of recovered waste heat due to its regenerative thermodynamic cycle. This gives the Stirling a greater advantage over internal combustion engines.

Recent investigations have been undertaken on the numerical and experimental analysis of Stirling engines powered by biomass combustion. An evaluation was carried out by Kuosa et al. [17] on the numerical evaluation of an alpha Stirling engine using the fouling factor to determine the effect of heat exchangers on Stirling engines for CHP application; the analysis considered the brake efficiency, output power and heat recovery to optimise the cleaning interval of the heat transfer surfaces when the cost model is combined with the performance model. Sato et al. [18] conducted a study on the use of a 55 kW_e_ Stirling engine in a CHP unit powered by wood powder. The combustion and inlet gas temperature were optimised to develop a cleaning process for hot ash, due to the ash fouling that was observed in the heat exchangers of the engine. The study showed that the introduction of a filter system reduced the heat transfer between the burner and Stirling engine, and the power output was affected negatively. Combustion tests were conducted by Nishiyama et al. [13] to analyse the efficiency and quality of wood powder combustion with the integration of a 55 kW_e_ Stirling engine. The air-to-fuel ratio effect on the output performance of the engine in relation to the hot end was highlighted. An experimental observation was conducted by Thiers et al. [19] on a commercial micro-CHP unit of a Sunmachine GmbH 1.5–3 kW_e_ alpha type Stirling engine powered by wood pellets with the purpose of developing a numerical model under transient conditions. The specified output performance of the manufacturer could not be achieved as the analysis resulted in high thermal losses, low power output and efficiency. Alfarawi et al. [20] presented an enhanced thermodynamic model of the gamma type Stirling engine operating on non-ideal adiabatic analysis. The model was validated against a Stirling engine prototype (ST05) and there was a good prediction in the shaft power, indicated power and thermal efficiency of the model operating at various conditions, including a parametric check on the influence of some of the engine’s parameters. Also the usage of stored cold energy of LN2 to optimise the power output of the engine was presented.

However, there has not been extensive research on the thermodynamic characteristics of the quasi steady state Stirling engine integrated with gasifier or combustor, with more detailed and accurate analyses of the output performance. In a previous work of this group [21], a small scale combustor was designed and commissioned; experimental tests were carried out on the combustion performance of human faeces, where characterisation of dry human faeces showed a high energy content of about 25 MJ/kg. In order to recover energy from the micro-combustor, including the conversion of heat losses into useful work, the integration process requires the design of a small scale Stirling engine to run at quasi steady state. Hence, there is a need for further investigation into the thermodynamic performance of the Stirling engine with biomass combustion, especially the heat exchangers, thermal losses, pressure drops, output power and efficiency of the engine.

This study examines the thermodynamic performance of the Stirling engine integrated with a self-sustaining sanitation technology for waste heat recovery and electrical power generation. The effect of different operating temperature profiles (including the heater, the cooler, the flue gas and working gas temperatures) on the thermal efficiency and power output are examined. In addition, the results are compared with the outputs from similar analyses on micro-CHP technologies with biomass combustion.

## 2 Methodology

### 2.1 Description of the NMT system

**Fig. 1:**
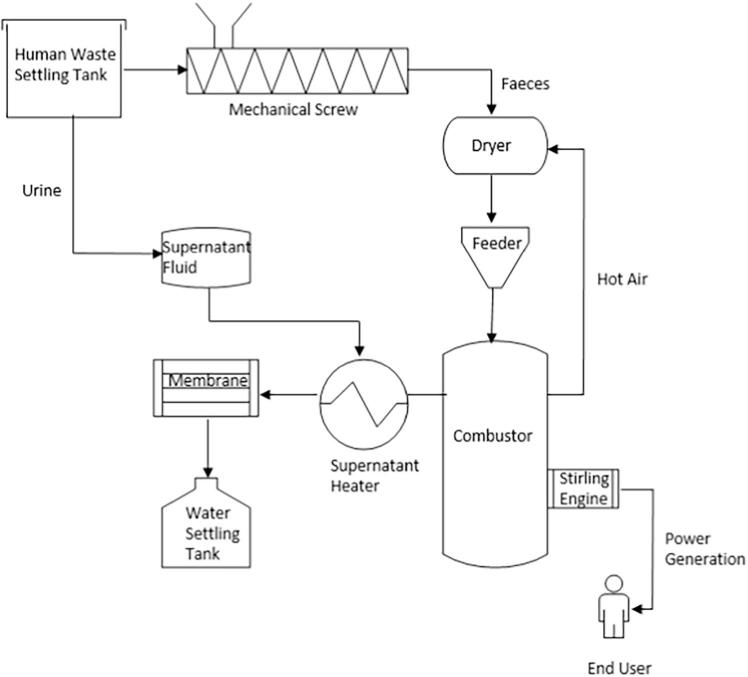
Flow process of the energy and water recovery system of the NMT.

The performance of the NMT system is defined by the collective operation of each integrated component, such as settling tank, mechanical screw, dryer, feeder, micro-combustor, membrane, water settling tank and Stirling engine [3], [21] as described in Fig. 1. In this system, human waste is received in the settling tank and separated into two streams: urine and faeces. The supernatant fluid (urine) is heated up by the supernatant heater before it is transported to the hollow-fibre membrane for purification. The resultant water is collected in the water settling tank. The faeces are transported by the mechanical screw to the dryer. Hot air is supplied to the dryer from the combustor to remove the moisture in the faeces before they proceed into the feeder. Faeces are transported into the combustor from the dryer with the aid of the feeder, which controls the feed rate of dried material into the combustor. The energy for the start-up of the combustor is supplied to the system via an air heater, which aids the increase of the operating temperature.

**Fig. 2:**
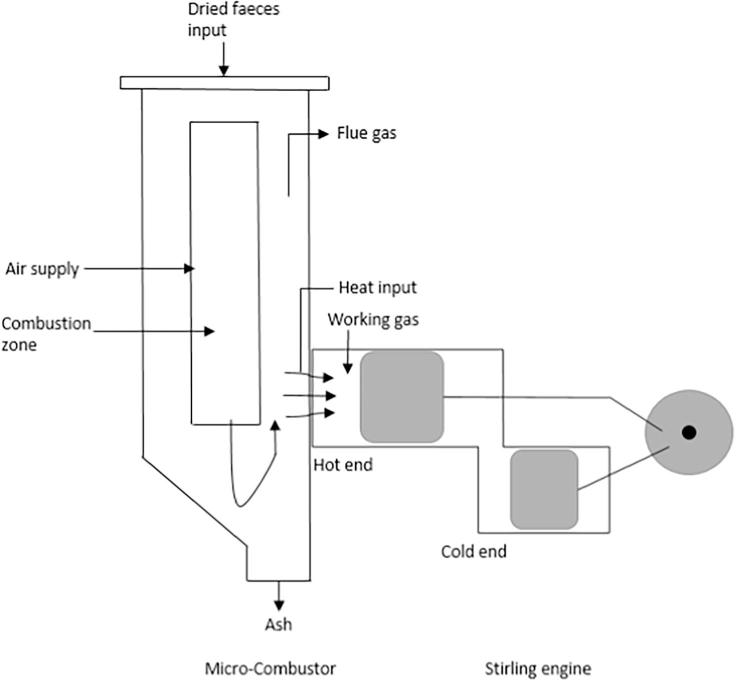
Schematic illustration of the integration of the micro-combustor with the Stirling engine showing heat fluxes through the combustor walls.

The exhaust temperature is expected to be about 400 °C with additional heat losses from the wall of the combustor to the environment on exposed surfaces. The Stirling engine is integrated into the combustor and connected to an alternator for electricity power generation. The product and by-products include clean water from the purified supernatant fluid, ash from burnt faecal material and exhaust gases. The Stirling engine is conceptualised to be integrated as the heat recovery system of the NMT unit. The hot-side of the Stirling engine, known as the heater, is assumed to be attached directly to the outer cylindrical wall of the combustor as depicted in Fig. 2, to recover waste heat that would otherwise be lost to the environment. This ensures that there is maximum heat surface area and heat transfer between the flue gas of the combustor and working gas in the Stirling engine. The recovered heat in the Stirling engine is then converted into electricity via an alternator. Therefore, the co-products of the NMT include electricity, clean water from the purified supernatant, and exhaust gases and ash from the combustion of faecal material.

### 2.2 Process description of the Stirling engine

**Fig. 3:**
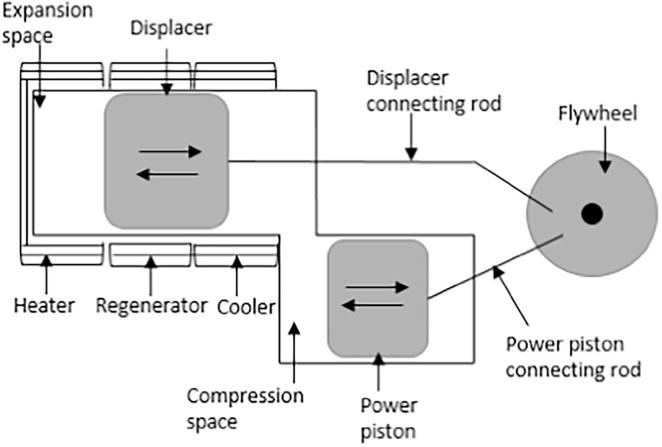
Schematic illustration of the gamma type Stirling engine.

The performance of the energy conversion unit proposed for the NMT has been previously described based on an experimental investigation on a bench-scale downdraft combustor test rig using faeces, wood biomass and simulant faeces [21]. This work focuses on the numerical investigation of the conceptualised heat recovery system of the NMT, using a quasi steady state model of the gamma type Stirling engine. Fig. 3 shows the schematic illustration of the gamma type Stirling engine used in this study.

The major factors that determine the Stirling engine performance are its thermal efficiency and power output. Other factors that contribute to the effective performance of the engine include: (i) mean pressure, which is the average pressure in the engine; (ii) working gas characteristics, such as high specific heat capacity, density, viscosity and thermal conductivity that vary with gas types, all of which contribute to the output performance of the engine; (iii) regenerator effectiveness, which defines the rate of heat absorbed from the working gas and the heat returned to the working gas during the oscillatory movement between the heat exchangers; (iv) work space temperature, which defines the temperature of the expansion and compression spaces; and (v) the swept volume, which defines the actual working gas volume in the work spaces. The higher the swept volume difference, the higher the power output from the pressure to volume (P-V) diagram.

### 2.3 Equations for numerical simulation

The heat transfer by conduction Q_cond_ is determined from Eq. (1). This rate determines the heat transfer from the flue gas to the working gas in the heater of the engine and plays a major role in the performance of the engine. An assumption of a constant heat loss of 30 °C with an increment of 10 °C is considered in the heat transfer from the flue gas temperature to the working gas via conduction as the temperature increases.

(1) $$Q_{\mathrm{cond}}=\mathrm{kA}\left(\frac{T_{w}-T_{\mathrm{wg}}}{d}\right)$$

where *k* is the thermal conductivity of the Stirling engine heater, and *A* is the surface area of the Stirling engine heater in contact with the combustor wall. T_w_ and T_wg_ are the temperature of the combustor wall (assumed to be the temperature of the heater wall) and temperature of the working gas respectively, and d is the thickness of the Stirling engine heater.

**Fig. 4:**
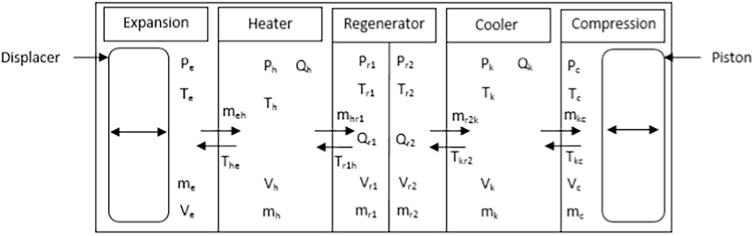
The control volumes in the Stirling engine.

The control volume in the Stirling engine is divided into six parts, i.e. the expansion space, heater space, two parts of the regenerator space, cooler space and the compression space (see Fig. 4). The analysis of the working gas volume in the two parts of the regenerator is important as the output and efficiency of the engine are determined by the performance of the regenerator. The numerical equations for the regenerator parts were applied to the thermodynamic modelling for accurate analysis of their performance in the engine.

Fig. 4 shows the six control volumes in the quasi steady state model of the Stirling engine used in this study. The pressure, temperature, mass flow and volumetric flow of the working gas across the work spaces and heat exchangers from the expansion space to the compression space, and the heat transfer in the heat exchangers, are exhibited in the diagram.

An ideal adiabatic model was applied for the numerical simulation of the gamma type Stirling engine. The equations used to determine the essential parameters for the numerical simulation can be found elsewhere [9], [22] and are given below**.**

Eqs. (2), (3) define the volume of expansion and compression spaces, respectively.

(2) $$V_{e}=V_{\mathrm{cle}}+\frac{V_{\mathrm{sd}}}{2}(1+\cos\theta )$$

where $V_{e}$ is the expansion space volume, $V_{\mathrm{cle}}$ is the clearance volume in the expansion space, $V_{\mathrm{sd}}$ is the swept volume of the displacer (all given in m^3^) and θ is the crank angle.

(3) $$V_{c}=V_{\mathrm{clc}}+\frac{V_{\mathrm{sp}}}{2}(1+\cos(\theta -\varphi )+\frac{V_{\mathrm{sd}}}{2}(1-\cos\theta )$$

where $V_{c}$ is the compression space volume, $V_{\mathrm{clc}}$ is the clearance volume in the compression space, $V_{\mathrm{sp}}$ is the swept volume of the piston (all in m^3^) and φ is the phase angle.

The derivatives of the gas temperatures in the compression and expansion spaces are calculated by means of Eqs. (4), (5).

(4) $${\mathrm{dT}}_{c}=T_{c}\left(\frac{\mathrm{dp}}{p}+\frac{\mathrm{dVc}}{\mathrm{Vc}}-\frac{\mathrm{dmc}}{\mathrm{mc}}\right)$$

where $T_{c}$ is the compression space temperature (°C), *P* is the pressure (bar) and $m_{c}$ is the mass of the working gas in the compression space (kg).

(5) $${\mathrm{dT}}_{e}=T_{e}\left(\frac{\mathrm{dp}}{p}+\frac{\mathrm{dVe}}{\mathrm{Ve}}-\frac{\mathrm{dme}}{\mathrm{me}}\right)$$

where $T_{e}$ is the expansion space temperature (°C) and $m_{e}$ is the mass of the working gas in the expansion space (kg).

The working gas temperature across the boundary between the heater and the expansion space is determined by Eqs. (6), (7):

(6) $$\text{If}\;{\overset{˙}{m}}_{\mathrm{he}}>0\text{,}\;T_{\mathrm{he}}=T_{h}$$

(7) $$\text{If}\;{\overset{˙}{m}}_{\mathrm{he}}⩽0\text{,}\;T_{\mathrm{he}}=T_{e}$$

where ${\overset{˙}{m}}_{\mathrm{he}}$ represents the mass flow rate from the heater to the expansion space (kg/s), $T_{\mathrm{he}}$ is the temperature of the stream flowing from the heater to the expansion space (°C), $T_{h}$ represents the temperature of the heater (°C) and $T_{e}$ is the temperature of the expansion space (°C).

The temperature of the gas stream flowing from the first part of the regenerator to the heater is described by Eqs. (8), (9):

(8) $$\text{If}\;{\overset{˙}{m}}_{r1h}>0\text{,}\;T_{r1h}=T_{\mathrm{rh}}$$

(9) $$\text{If}\;{\overset{˙}{m}}_{r1h}⩽0\text{,}\;T_{r1h}=T_{h}$$

where ${\overset{˙}{m}}_{r1h}$ is the mass flow rate from the first part of the regenerator to the heater (kg/s), $T_{r1h}$ is the temperature of the stream flowing from the first part of the regenerator to the heater (°C), and $T_{\mathrm{rh}}$ is the temperature between the first part of the regenerator space and the heater (°C).

The boundary condition for mass flow rates from the cooler to the second part of the regenerator is defined by Eqs. (10), (11).

(10) $$\text{If}\;{\overset{˙}{m}}_{\mathrm{kr}2}>0\text{,}T_{\mathrm{kr}2}=T_{k}$$

(11) $$\text{If}\;{\overset{˙}{m}}_{\mathrm{kr}2}⩽0\text{,}T_{\mathrm{kr}2}=T_{\mathrm{rk}}$$

where ${\overset{˙}{m}}_{\mathrm{kr}2}$ represents the mass flow rate from the cooler to the second part of the regenerator (kg/s), $T_{\mathrm{kr}2}$ is the temperature of the stream flowing from the cooler to the second part of the regenerator (°C), $T_{k}$ is the temperature of the cooler space, (°C) and $T_{\mathrm{rk}}$ represents the temperature between the second part of the regenerator and the cooler (°C).

The mass flow rate for the boundary between the compression space to the cooler is given by Eqs. (12), (13).

(12) $$\text{If}\;{\overset{˙}{m}}_{\mathrm{ck}}>0\text{,}T_{\mathrm{ck}}=T_{c}$$

(13) $$\text{If}\;{\overset{˙}{m}}_{\mathrm{ck}}⩽0\text{,}T_{\mathrm{ck}}=T_{k}$$

$${\overset{˙}{m}}_{\mathrm{ck}}$$

represents the mass flow rate from the compression space to the cooler (kg/s), $T_{\mathrm{ck}}$ is the temperature between the compression space and the cooler (°C), and $T_{c}$ represents the temperature of the compression space (°C).

The application of the energy conservation equation to determine the control volumes can be defined by means of Eqs. (14), (15), (16).

(14) $$C_{\mathrm{av}}\frac{d(m_{c}T_{c})}{\mathrm{dt}}=-{\overset{˙}{m}}_{\mathrm{ck}}C_{\mathrm{ap}}T_{\mathrm{ck}}-\frac{{\mathrm{dW}}_{c}}{\mathrm{dt}}$$

(15) $$C_{\mathrm{av}}\frac{{\mathrm{dm}}_{c}T_{c}}{\mathrm{dt}}={\mathrm{dQ}}_{c}-{\mathrm{dQ}}_{\mathrm{kdiss}}+{\overset{˙}{m}}_{\mathrm{ck}}C_{\mathrm{ap}}T_{\mathrm{ck}}-{\overset{˙}{m}}_{\mathrm{cr}1}C_{\mathrm{ap}}T_{\mathrm{cr}1}$$

(16) $$C_{\mathrm{av}}\frac{d(m_{r1}T_{r1})}{\mathrm{dt}}={\mathrm{dQ}}_{r1}-{\mathrm{dQ}}_{r1\mathrm{diss}}+{\overset{˙}{m}}_{\mathrm{cr}1}C_{\mathrm{ap}}T_{\mathrm{cr}1}-{\overset{˙}{m}}_{r1\_r2}C_{\mathrm{ap}}T_{r1\_r2}$$

The regenerator is divided into two parts to investigate its performance based on its proximity to the heater and cooler. The parts of the regenerator are derived as Eqs. (17), (18), (19), (20).

(17) $$C_{\mathrm{av}}\frac{{\mathrm{dm}}_{r(1)}T_{r(1)}}{\mathrm{dt}}={\mathrm{dQ}}_{r(1)}-{\mathrm{dQ}}_{r(1)\mathrm{diss}}+{\overset{˙}{m}}_{r(1)r(1)}C_{\mathrm{ap}}T_{r(1)r(1)}-{\overset{˙}{m}}_{r(1)r(1)}C_{\mathrm{ap}}T_{r(1)r(1)}$$

(18) $$C_{\mathrm{av}}\frac{{\mathrm{dm}}_{r(2)}T_{r(2)}}{\mathrm{dt}}={\mathrm{dQ}}_{r(2)}-{\mathrm{dQ}}_{r(2)\mathrm{diss}}+{\overset{˙}{m}}_{r(2-1)r(2)}C_{\mathrm{ap}}T_{r(2-1)r(2)}-{\overset{˙}{m}}_{r(2)r(2+1)}C_{\mathrm{ap}}T_{r(2)r(2+1)}$$

(19) $$C_{\mathrm{av}}\frac{d(m_{h}T_{h})}{\mathrm{dt}}={\mathrm{dQ}}_{h}-{\mathrm{dQ}}_{\mathrm{hdiss}}+{\overset{˙}{m}}_{r2h}C_{\mathrm{ap}}T_{r2h}-{\overset{˙}{m}}_{\mathrm{he}}C_{\mathrm{ap}}T_{\mathrm{he}}$$

(20) $$C_{\mathrm{av}}\frac{\left({\mathrm{dm}}_{e}T_{e}\right)}{\mathrm{dt}}={\overset{˙}{m}}_{\mathrm{he}}C_{p}T_{\mathrm{he}}-\frac{{\mathrm{dW}}_{e}}{\mathrm{dt}}$$

In the above equations, $m_{k}\text{,}$ $m_{r}$ and $m_{h}$ are the mass of working gas in the cooler, regenerator and heater, respectively (kg), $W_{c}$ is the work in the compression space (J), $W_{e}$ is the work in the expansion space (J), $Q_{\mathrm{cool}}$, $Q_{\mathrm{reg}}$, $Q_{\mathrm{heat}}$ are the heat transfer in the cooler, regenerator, and heater, respectively (W), $Q_{\mathrm{kdiss}}$ is the heat dissipation loss due to friction in the cooler (W), $Q_{r(j)\mathrm{diss}}$ is the heat dissipation loss due to friction in the two parts of the regenerator (W), $Q_{\mathrm{hdiss}}$ is the heat dissipation loss due to friction in the heater (W). The conservation of energy for each control volume is obtained by using the ideal gas state equation of the working gas (Eq. (21)), and the equations for the work done in the compression and expansion spaces, Eqs. (22), (23) respectively.

(21) PV=mRT

where *P* is the pressure (atm), *V* volume (m^3^), *m* mass of helium (working gas) (kg), *R* specific gas constant for helium (in atm L/ kg °C) and *T* temperature of the working gas (°C).

(22) $$\frac{{\mathrm{dW}}_{c}}{\mathrm{dt}}=P_{c}\frac{{\mathrm{dV}}_{c}}{\mathrm{dt}}$$

(23) $$\frac{{\mathrm{dW}}_{e}}{\mathrm{dt}}=P_{e}\frac{{\mathrm{dV}}_{e}}{\mathrm{dt}}$$

Eqs. (24), (25) are used to calculate the rate of heat transfer in the heat exchangers:

(24) $${\mathrm{dQ}}_{\mathrm{heat}}=h_{h}A_{\mathrm{surfh}}(T_{\mathrm{wh}}-T_{h})-{\mathrm{dQ}}_{\mathrm{hintl}}$$

where ${\mathrm{dQ}}_{\mathrm{hintl}}$ is the heat loss due to the heat conduction from the part of the heater with the higher temperature to the part of the heater with the lower temperature (W), $h_{h}$ is the heat transfer coefficient of the heater (W/m^2^ K), $A_{\mathrm{surfh}}$ is the surface area of the heater (m^2^), and $T_{\mathrm{wh}}$ is the temperature of the heater wall (°C).

(25) $${\mathrm{dQ}}_{\mathrm{cool}}=h_{k}A_{\mathrm{surfk}}(T_{\mathrm{wk}}-T_{k})-{\mathrm{dQ}}_{\mathrm{kintl}}$$

where ${\mathrm{dQ}}_{\mathrm{kintl}}$ is the heat loss due to the heat conduction from the part of the cooler with the higher temperature to the part of the cooler with the lower temperature (W), $h_{k}$ is the heat transfer coefficient of the cooler (W/m^2^ K), $A_{\mathrm{surfk}}$ is the surface area of the cooler (m^2^), and $T_{\mathrm{wk}}$ is the temperature of the cooler wall (°C).

The rate of heat transfer in the two parts of the regenerator is determined by means of Eq. (26).

(26) $${\mathrm{dQ}}_{\mathrm{reg}}=\varepsilon h_{r(j)}A_{\mathrm{surfr}(j)}(T_{m(j)}-T_{r(j)})-{\mathrm{dQ}}_{r(j)\mathrm{intl}}$$

where ε is emissivity, $h_{r}$ is the heat transfer coefficent of the regenerator (W/m^2^ K) and $A_{\mathrm{surfr}(j)}$ is the free surface area of the regenerator (m^2^), ${\mathrm{dQ}}_{\mathrm{rintl}}$ is the heat loss due to the heat conduction from the part of the regenerator with the higher temperature to the part of the regenerator with the lower temperature (W), and (j) represents each part of the regenerator matrix.

Eqs. (27), (28), (29) are used to determine the one-dimensional heat conduction loss along the length of the heat exchanger for the cooler, heater and the regenerator parts, respectively.

(27) $${\mathrm{dQ}}_{\mathrm{kintl}}=\frac{k_{k}A_{k}}{l_{k}(T_{\mathrm{kr}}-T_{\mathrm{ck}})}$$

where $k_{k}$ represents the thermal conductivity of the cooler (W/m K), $A_{k}$ represents the cross-sectional area of the cooler (m^2^) and $l_{k}$ is the length of the cooler (m), $T_{\mathrm{kr}}$ represents the temperature between the cooler and regenerator (°C), and $T_{\mathrm{ck}}$ represents the temperature between the compression space and cooler (°C).

(28) $${\mathrm{dQ}}_{\mathrm{hintl}}=\frac{k_{h}A_{h}}{l_{h}(T_{\mathrm{he}}-T_{\mathrm{rh}})}$$

where $k_{h}$ represents the thermal conductivity of the heater (W/m K), $A_{h}$ the cross-sectional area of the heater (m^2^) and $l_{h}$ represents the length of the heater (m), $T_{\mathrm{rh}}$ is the temperature between the regenerator and the heater (°C) and $T_{\mathrm{he}}$ is the temperature between the heater and expansion space (°C).

(29) $${\mathrm{dQ}}_{\mathrm{rintl}}=\frac{k_{r}A_{r}}{l_{r}(T_{\mathrm{rr}}-T_{\mathrm{kr}})}$$

where $k_{r}$ is the thermal conductivity of the regenerator (W/m K), $A_{r}$ is the cross-sectional area of the regenerator (m^2^) and $l_{r}$ is the length of the regenerator (m), $T_{\mathrm{kr}}$ is the temperature between the cooler and regenerator (°C), and $T_{\mathrm{rr}}$ is the temperature between the regenerator parts (°C).

For pressure drop in the heat exchangers, Eq. (30) given in [23] is employed.

(30) $$\Delta p=-\frac{2f_{c}\mu \mathrm{UV}}{A_{f}h_{d}^{2}}$$

where $f_{c}$ represents the Reynolds friction coefficient; μ represents the dynamic viscosity of the working gas (Pa.s); U represents fluid velocity (m/s); V represents the volume (m^3^); $A_{f}$ the free surface area (m^2^) and $h_{d}$ the hydraulic diameter (m).

The shuttle loss accounted for in this study is given by Eq. (31).

(31) $${\mathrm{dQ}}_{\mathrm{sht}}=\frac{0.4X_{d}^{2}k_{f}d_{d}}{\Delta {\mathrm{gpL}}_{d}}$$

where $X_{d}$ represents the displacer stroke (m), $k_{f}$ is the thermal conductivity of the working gas (W/m K), $d_{d}$ represents the diameter of the displacer (m) and $l_{d}$ is the length of the displacer (m).

The derivative for the total pressure in the engine is obtained by summing all the energy equations and losses as given by Eq. (32).

(32) $$\frac{\mathrm{dp}}{\mathrm{dt}}=\frac{1}{C_{\mathrm{av}}V_{t}}\left(R({\mathrm{dQ}}_{h}+\sum {\mathrm{dQ}}_{r}+{\mathrm{dQ}}_{c}-\sum {\mathrm{dQ}}_{\mathrm{diss}}-{\mathrm{dQ}}_{\mathrm{intl}}-{\mathrm{dQ}}_{\mathrm{shtl}})\right)-C_{\mathrm{ap}}\left(P_{e}\frac{{\mathrm{dV}}_{e}}{\mathrm{dt}}+P_{c}\frac{{\mathrm{dV}}_{c}}{\mathrm{dt}}\right)$$

The indicated work in the cycle and the indicated power of the engine are defined by Eqs. (33), (34) respectively:

(33) $$W_{\mathrm{in}}=\int_{0}^{t}\left(P_{e}\frac{{\mathrm{dV}}_{e}}{\mathrm{dt}}+P_{c}\frac{{\mathrm{dV}}_{c}}{\mathrm{dt}}\right)$$

(34) $$P_{\mathrm{in}}=W_{\mathrm{in}}f$$

*W* is the total indicated work done and *f* is the frequency.

MATLAB was employed to write the codes for the numerical simulation using the mathematical equations stated and to determine the output performance and thermodynamic characteristics. The initial input parameters for the Stirling engine were obtained from the design specifications listed in Table 1. The model is based on the concept of the working gas inside the Stirling engine heater being heated through conduction by the hot flue gas leaving the combustor. The heat transfer by conduction is calculated, and the heater temperature is provided as an input. The set of differential equations were solved and the unknown function that satisfies the initial conditions of the differential equations was the objective, which was computed for each of the output parameters.

**Table 1:** Characteristics of the 1 kW gamma Stirling engine used in this study [24]

Engine Data	Value
*General*	
Maximum pressure (bar)	58
Heater temperature (°C)	390
Cooler temperature (°C)	50
Phase angle (degree)	90
Working gas	Helium
Stroke of displacer (m)	0.025
Stroke of piston (m)	0.025

*Geometry*	
Heater diameter (m)	0.03
Regenerator diameter (m)	0.08
Cooler diameter (m)	0.0012
Piston diameter (m)	0.062
Displacer diameter (m)	0.062
Length of heater (m)	0.24
Length of regenerator (m)	0.04
Length of cooler (m)	0.05
Regenerator porosity (%)	0.75
Thermal conductivity of heater (W m^−1^ K^−1^)	36.2

**Fig. 5:**
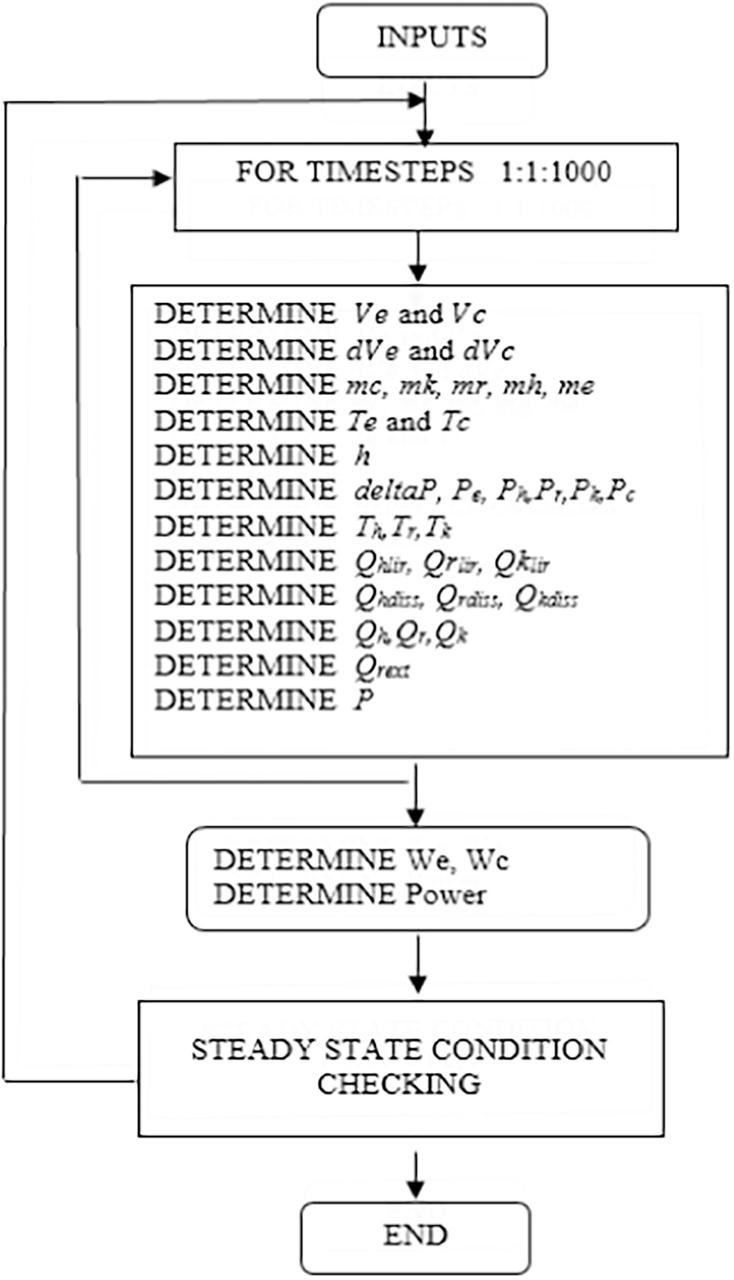
Flow chart of the numerical simulation.

The equations in the operating system were integrated through complete cycles to determine the pressure, displacement and velocity of the piston and displacer, the pressure drop and heat losses, and the volume of the expansion and compression spaces until the steady state operation of the engine was achieved. The heat exchangers (heater, regenerator and cooler) were modelled with an infinite surface area similar to the ideal Stirling engine, and the steady state condition was achieved when the temperature of the expansion and compression space at the beginning of the cycle was the same as the temperature at the end of the cycle. The consecutive process of the computation for the numerical procedure is described by the flow chart in Fig. 5.

### 2.4 Assumptions for the gamma type Stirling engine mathematical model

The assumptions listed next were made to obtain the mathematical model of the Stirling engine:

1. The working gas is an ideal gas.
2. The heat losses in the Stirling engine are accounted for in the simulation.
3. Leakage of working gas is not expected to occur and is not considered.
4. The temperature of the working gas changes with time in the surrounding wall in the engine compartment.
5. The regenerator temperature is equal to the average temperature of the heater and cooler.
6. The Stirling engine is in a steady state operation.
7. The pressure drops of the working gas in the heat exchangers are considered.

## 3 Results and discussion

### 3.1 Model validation

The quasi steady state model developed in this study was validated with the experimental results from the gamma type Stirling engine reported by Gheith et al. [25].

In order to validate the model the brake power of the Stirling engine was calculated by first establishing the total torque on the crankshaft. Equations obtained from [26], [27] were used for this calculation. The torque (*Tq*) from the piston and displacer is determined with Eq. (35);

(35) $$\mathrm{Tq}=\frac{1}{2}{\overset{˙}{\beta }}^{2}I^{\prime }(\beta )+g(\beta )+Q(t\text{,}\beta )$$

where $I^{\prime }(\beta )$ represents the rate of change of inertia, g(β) represents the torque from gravity and Q(t,β) represents the loading torque on the piston. To account for the mechanical loss in the engine due to friction (${\mathrm{Tq}}_{b})$, Eq. (36) is used.

(36) $${\mathrm{Tq}}_{b}=\mu_{c-b}F_{a}\frac{B_{d}}{2}$$

where $\mu_{c-b}$ represents the coefficient of friction in the ball bearing, $F_{a}$ represents the absolute force acting on the bearing (N) and $B_{d}$ represents the bearing diameter (m).

The cyclic brake work is determined with Eq. (37);

(37) $$W_{\mathrm{br}}=\oint \left(\frac{{\mathrm{dW}}_{\mathrm{br}}}{\mathrm{dt}}\right)\mathrm{dt}=\int_{0}^{t}({\mathrm{Tq}}_{\tau }-{\mathrm{Tq}}_{b})\frac{d\theta }{\mathrm{dt}}\mathrm{dt}$$

And the cyclic brake power is determined with Eq. (38)

(38) $$P_{\mathrm{br}}=W_{\mathrm{br}}f$$

The values of the parameters used as input to the model for validation are presented in Table 2. The experimental and model results were compared at a constant heater temperature of 400 °C and different charge pressures of 3, 5 and 8 bar, as shown in Table 3. The Root Mean Square (RMS) error was calculated to determine the error margin through Eq. (39). The results for output power obtained from the model and presented in Gheith et al. [25] were similar, with an RMS error lower than 0.050% in all cases.

(39) $$\mathrm{RMS}=\sqrt{\frac{{\left(\mathrm{REF}-\mathrm{MOD}\right)}^{2}}{n}}$$

where REF is the value obtained from a previous study [25], MOD the value obtained from the present study, and n the number of data.

**Table 2:** Input parameters of the gamma Stirling engine for model validation (data collected from Gheith et al. [25]).

Engine Data	Value
*General*	
Mean Pressure (bar)	10
Heater Temperature (°C)	500
Cooler Temperature (°C)	15
Phase angle (degree)	90
Working gas	Air

*Geometry*	
Compression space diameter (m)	0.08
Compression space height (m)	0.145
Expansion space diameter (m)	0.095
Expansion space height (m)	0.120
Regenerator outer diameter (m)	0.134
Regenerator inner diameter (m)	0.098
Regenerator height (m)	0.05

*Regenerator Material – Stainless Steel 304L*	
Porosity (%)	0.9
Density (g m^−3^)	7.850
Thermal capacity (J kg^−1^ K^−1^)	477
Thermal conductivity (W m^−1^ K^−1^)	26

**Table 3:** Results from the validation of the numerical model of the gamma Stirling engine against the experimental output.

Regenerator material	Heater Temperature	Charge Pressure	Brake Power (W) (Experiment)	Brake power (W) (Simulation)	RMS Error (%)
Stainless Steel	400	3	150	146	0.028
		5	275	269	0.042
		8	308	303	0.050

### 3.2 Stirling engine performance

Fig. 6a, Fig. 6b, Fig. 7a, Fig. 7b, Fig. 8a, Fig. 8b, Fig. 9a, Fig. 9b, Fig. 10a, Fig. 10b illustrate the thermodynamic performance of the Stirling engine at a heater temperature of 390 °C and cooler temperature of 50 °C.

**Fig. 6a:**
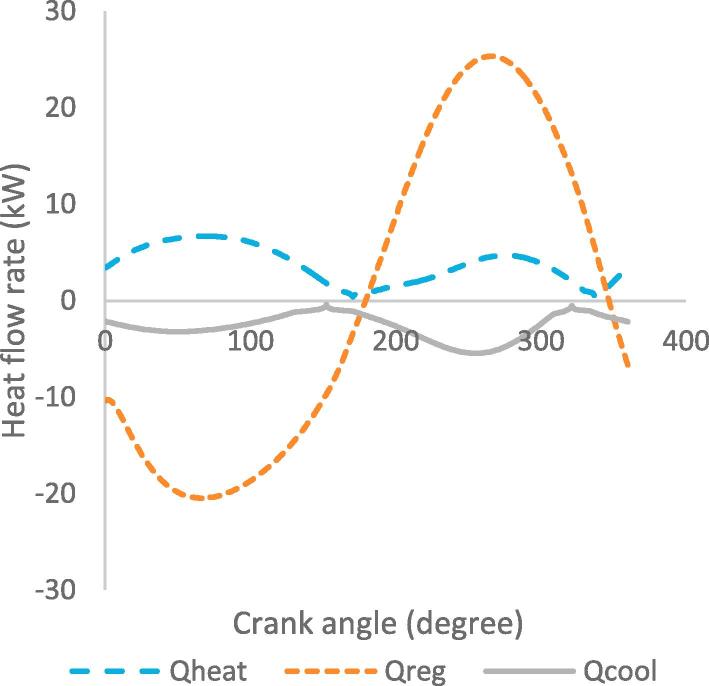
Heat transfer in the heat exchangers.

**Fig. 6b:**
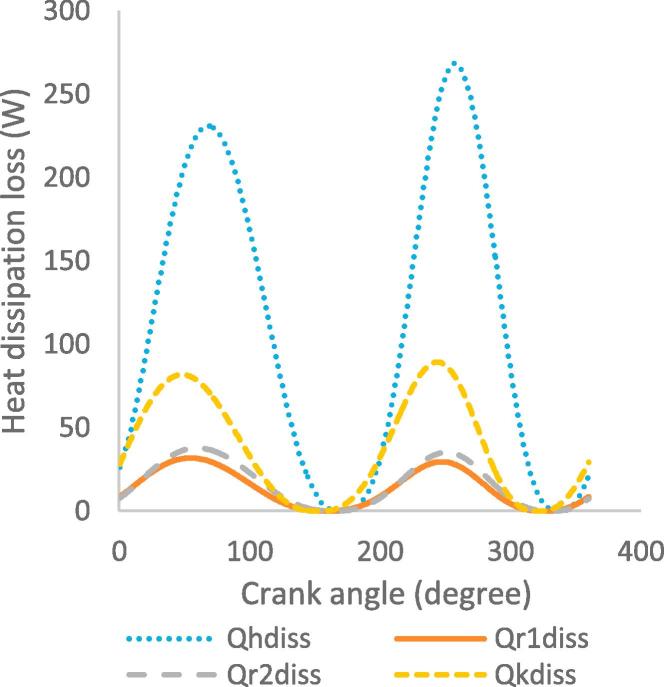
Heat dissipation loss in heat exchangers.

**Fig. 7a:**
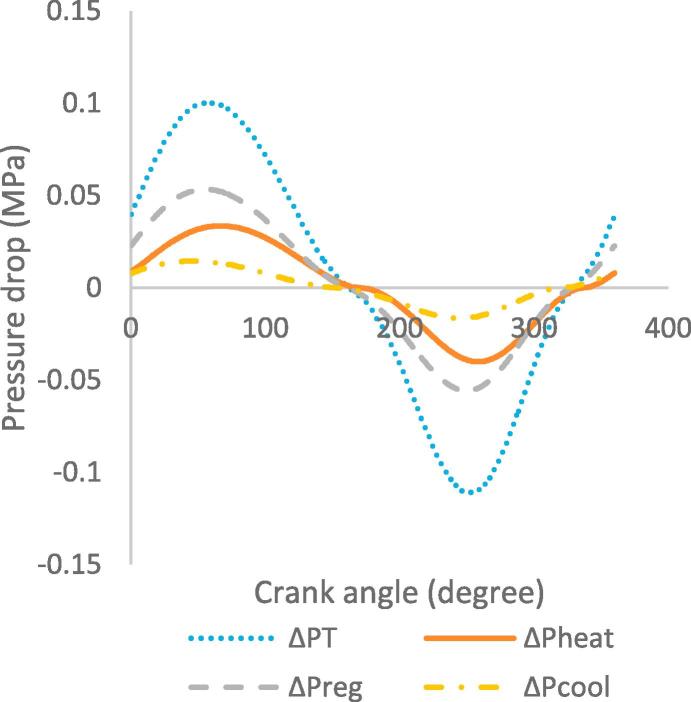
Pressure drop in the heat exchangers, workspaces and heat exchangers.

**Fig. 7b:**
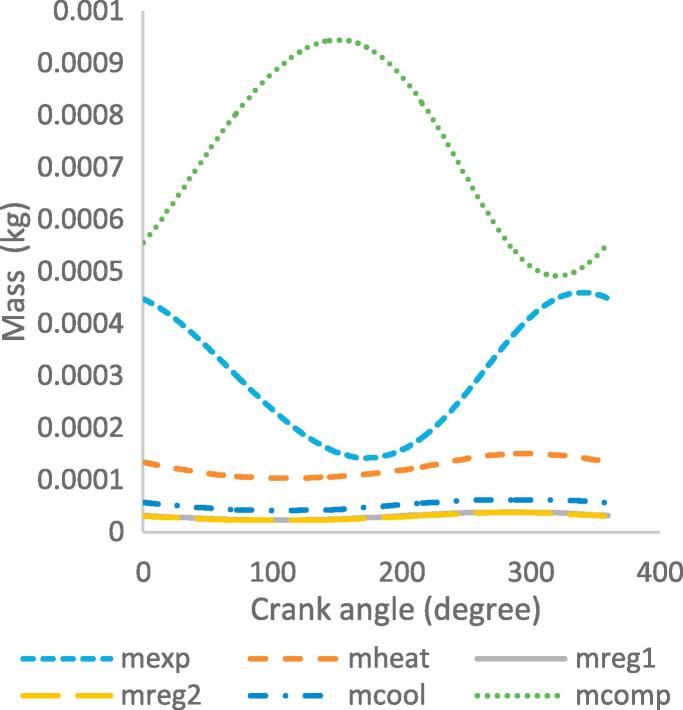
Mass of the working gas in the workspaces and heat exchangers.

**Fig. 8a:**
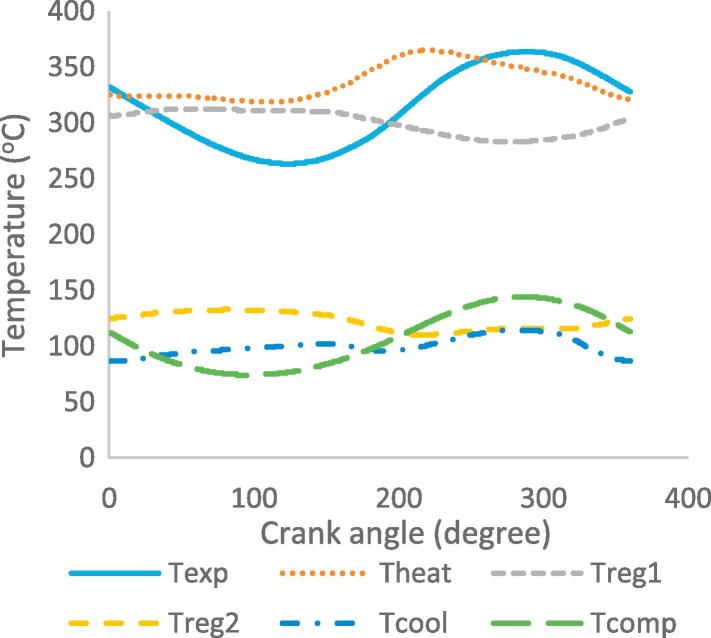
Temperature of the workspaces and heat exchangers at steady state.

**Fig. 8b:**
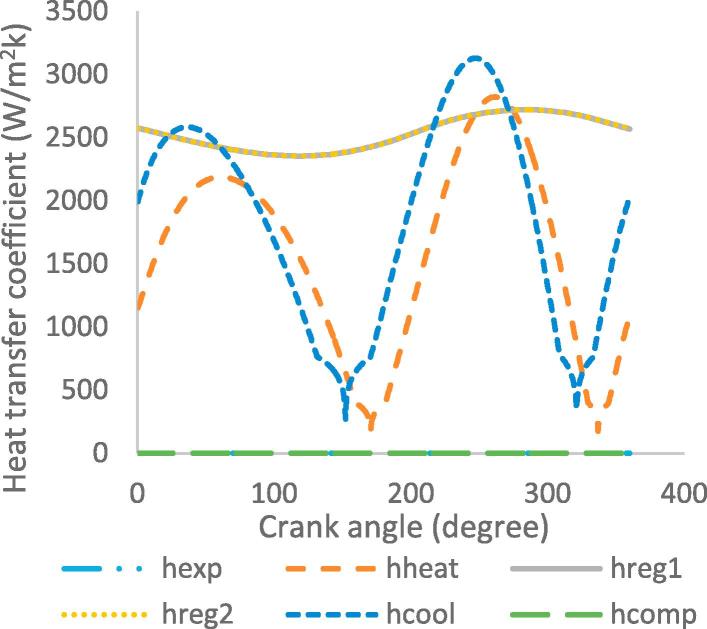
Heat transfer coefficient in the workspaces and heat exchangers.

**Fig. 9a:**
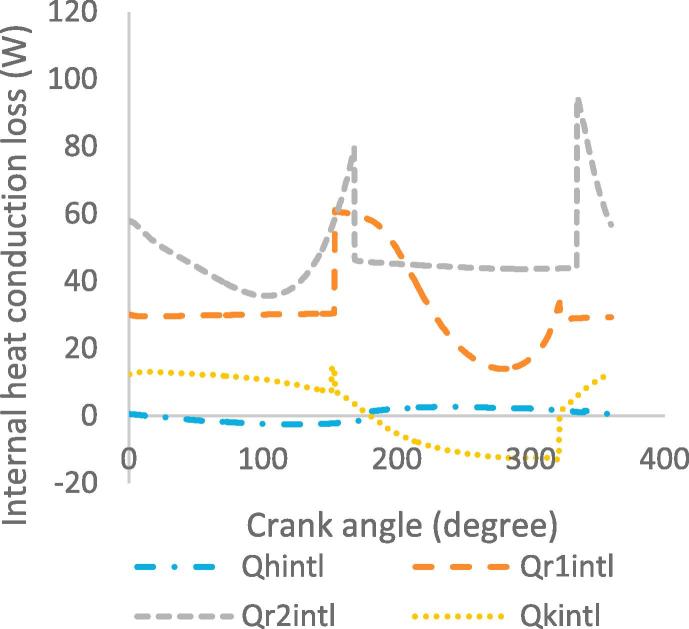
Internal heat conduction loss.

**Fig. 9b:**
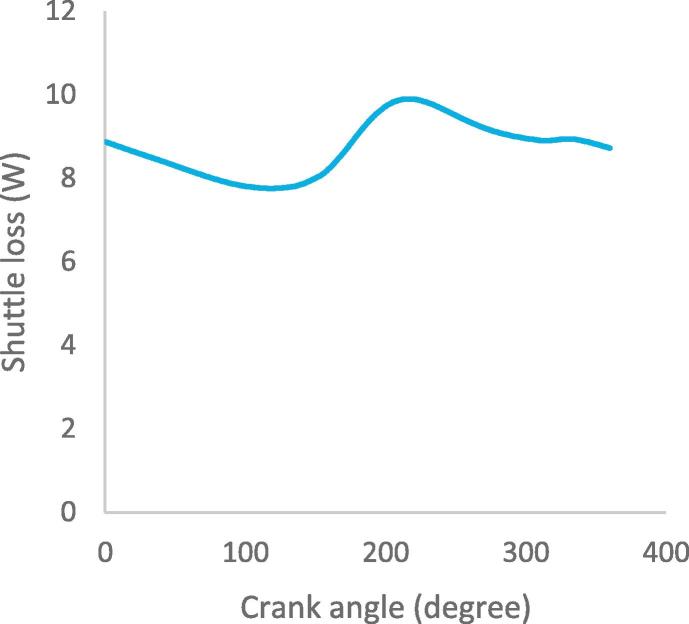
Shuttle loss in heat exchangers.

**Fig. 10a:**
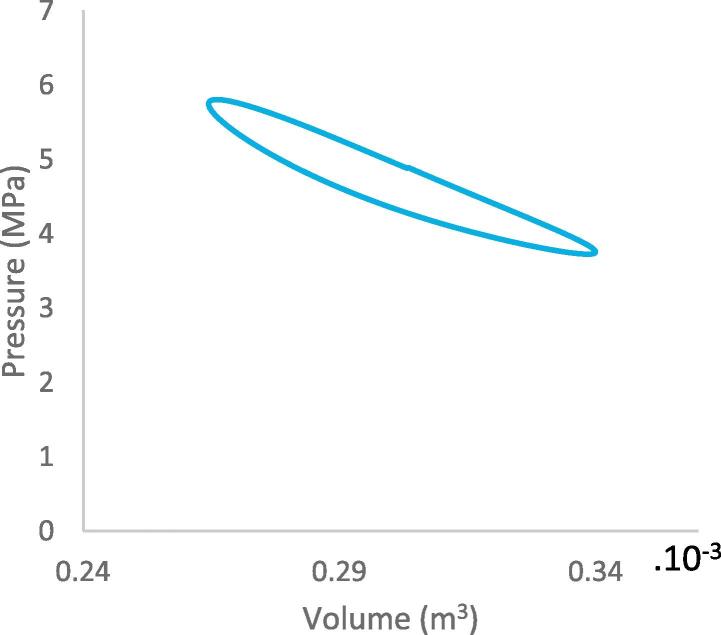
P-V diagram of the work spaces.

**Fig. 10b:**
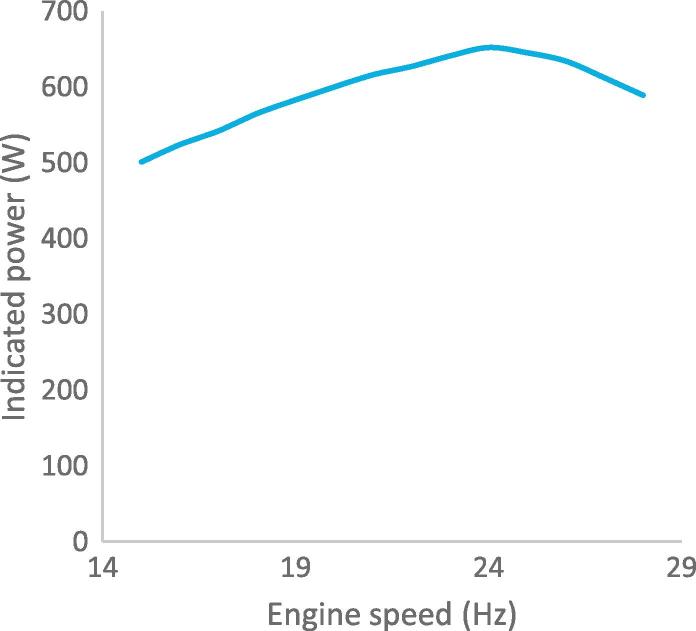
Power output against engine speed.

Fig. 6a shows the flow rate of heat from the heater to regenerator and cooler as a function of the crank angle. The maximum and minimum heat flow rates correspond to the instances when the gas velocity is at the highest and lowest in the cycle. The variations in the heat flow rate in the heat exchangers occur when the displacer and pistons are at the top and bottom dead centre positions. The maximum heat flow rate in the heater is 6.8 kW at 65°Crank angle, and maximum heat flow rate in the cooler is −0.91 kW at 322°Crank angle. At 274°Crank angle, the regenerator exhibits the maximum heat flow rate of 25.3 kW. The regenerator exhibits the highest values of the heat flow rate due to its effectiveness absorbing and rejecting heat from the working gas in the regenerator chamber.

The heat dissipation loss in the heat exchangers, as shown in Fig. 6b, indicates that the heater has the highest heat dissipation rate of 266 W at crank angle of 265°, while the cooler presents a lower dissipation rate of 88.2 W at crank angle of 250°. These results show that the heat flow rate of the working gas in the heat exchangers varies with the displacement of the working gas volume.

Fig. 7a illustrates the relationship between the pressure drop in the heat exchangers and the crank angle at steady state. The highest pressure drop is observed in the regenerator at a value of 0.051 MPa at crank angle of 55°. The heater and cooler present maximum pressure drops of 0.033 MPa at crank angle of 57° and 0.015 MPa at crank angle of 37° respectively; the difference in the crank angle is as a result of the displacer movement towards the top dead centre. The maximum total pressure drop, which is calculated as the addition of pressure drops in the regenerator, heater and cooler, is approximately 0.099 MPa. The highest pressure drop in the regenerator indicates hydraulic resistance and can have a measurable effect on the output power and efficiency of the Stirling engine [28]. Therefore, the detailed design of the heat exchangers, especially the regenerator should be considered and investigated. From Fig. 7a, it can be seen that the values of the pressure drop at the beginning of the cycle are the same as those at the end of the cycle, which means the engine is in a steady state operation. The pressure drop varies in the heat exchangers (heater, regenerator and cooler) from maximum to minimum values in the cycle and this is affected by the oscillatory displacement of the working gas.

The mass of the working gas as a function of the crank angle is illustrated in Fig. 7b. The compression space exhibited the highest mass of the working gas volume due to the pressure increment during the compression of the working gas when the displacer moves towards the bottom dead centre and the piston moves towards the top dead centre. The expansion space exhibits a lower mass of the working gas due to the expansion as the displacer moves towards the bottom dead centre. At the beginning of a cycle, the mass in the compression and expansion spaces increases and decreases respectively. This is due to the relative motions of the piston and displacer as well as the temperature, volume and pressure of the working gas in the work spaces. The heat exchangers exhibit the lowest mass values, with the mass in the heater and cooler being higher than that in the regenerator. This is related to the displacement of the working gas and proximity to the expansion and compression spaces as the working gas moves across the chambers.

The temperature of the work spaces and heat exchangers is shown in Fig. 8a. The working gas in the heater and the expansion space exhibit maximum temperatures of 365 °C and 364 °C at crank angles of 220° and 288° respectively. This shows that the heat loss between the expansion space and heater is negligible. The regenerator is divided into two parts and it can be seen that the part of the regenerator closest to the heater has a higher temperature than the part of the regenerator closest to the cooler; the difference in temperature is, therefore, affected by the proximity of the regenerator to the heater and cooler. The working gas in the compression space presents a maximum temperature of 144 °C at crank angle of 288°, and the cooler temperature of 114 °C at crank angle of 297°. The temperature difference is due to the compressive force on the working gas by the displacer and piston, thereby increasing the pressure and temperature of the working gas in the compression space before moving towards the cooler, which reduces the temperature.

It is worth noting that from the steady state condition criteria in the numerical simulation, the values of the expansion and compression temperature at the beginning and end of the cycle must be the same. Fig. 8a shows that this condition is satisfied. The temperature difference between work spaces (expansion and compression) depends on the heater and cooler, due to their location in the engine geometry. The expansion space and heater temperatures depend on the temperature of the working gas; and the higher the working gas temperature, the higher the output power and efficiency of the Stirling engine.

The heat transfer coefficient in the Stirling engine against the crank angle is shown in Fig. 8b. The variation in the cycle shows the heater and cooler have the highest heat transfer coefficients. The heater exhibits a maximum coefficient of 2.565 kW/m^2^ K at crank angle of 270°, which shows a high transfer of heat to the working gas. Each part of the regenerator shows a maximum heat transfer coefficient of 2.716 kW/m^2^ K at crank angle of 274°, which indicates the regenerator is effective in the transfer and removal of heat from the working gas. The cooler exhibits a maximum heat transfer coefficient of 3.101 kW/m^2^ K at crank angle of 252°. This shows that the cooler considerably dissipates the heat from the working gas before it is transferred to the compression space. The expansion and compression spaces exhibit a very low heat transfer coefficient, as work is being done on the working gas in the spaces.

Fig. 9a illustrates the variation in the internal heat conduction losses in the heat exchangers as a function of the crank angle. The part of the regenerator closest to the cooler shows a maximum heat loss of 92 W at crank angle of 336°, while the part closest to the heater shows a maximum heat loss of 60 W at crank angle of 155°. The noticeable difference in the internal heat conduction losses between the parts of the regenerator is due to their proximity effect to the heater and cooler. The maximum heat loss in the heater and cooler are 2.5 W and 14.2 W at crank angles of 226° and 153° respectively. The rapid drop in the internal heat conduction loss in the heat exchangers is due to the working gas movement in the cylinder with respect to the position of the displacer as it approaches the bottom dead centre. In Fig. 9b, the maximum shuttle loss during the displacer reciprocating motion is 10 W at steady state in the cycle; this is the heat transferred to the cold working space of the cylinder from the hot space when the displacer is at the top dead centre in the cylinder.

The results obtained through the model confirm that the working gas temperature can greatly affect the performance of the Stirling engine. The amount of heat transfer from the flue gas to the working gas depends on the position of the Stirling engine on the outer cylindrical surface of the combustor. The model in this study was built on the assumption that the Stirling engine is positioned close to where the flue gas is leaving the combustion chamber and hence at the maximum temperature, in order to ensure maximum temperature gradient between the flue gas and working fluid via conduction. Cardozo et al. [29] discussed the importance of the position of the Stirling engine in relation to the combustor, in order to absorb as much heat as possible from the combustor and improve the total output performance. Beyond this thermodynamic analysis, all these factors need to be considered when the combustor and Stirling engine are physically integrated in future experimental campaigns.

Fig. 10a shows the P-V diagram of the work spaces. This diagram represents the relationship between the pressure and change in the working gas volume of the Stirling engine. The operating pressure has a maximum of 5.78 MPa and minimum of 3.34 MPa, the pressure ratio is 1.72. There is a difference between the maximum and minimum pressure in the expansion and compression spaces. This is as a result of the pressure drop in the heat exchangers when the working gas flows through their chambers. The area enclosed by the curves illustrates the positive work done in the expansion space and the negative work done in the compression space. The calculated area of the P-V diagram of the expansion space indicates the positive work done, while the calculated area of the compression space indicates the negative work done. The net indicated power calculated from the difference of the P-V diagrams is therefore 652 W, at thermal efficiency of 17.81% and engine speed of 1431 rpm. The enclosed areas in the P-V diagram formed are due to the dead volume in the work space and non-sinusoidal motion of the drive mechanism in contrast to the ideal Stirling cycle. The power output against the engine speed is shown in Fig. 10b. It can be observed that the indicated power rises to 652 W at a maximum speed of 1431 rpm (23.85 Hz), and the power output reduces with further increase in the engine speed. This is as a result of frictional losses in the crankshaft of the engine.

Based on the requirement of maximum heat flow rate, the results in Fig. 6a, Fig. 6b, Fig. 7a, Fig. 7b, Fig. 8a, Fig. 8b, Fig. 9a, Fig. 9b, Fig. 10a, Fig. 10b therefore imply that the engine is best operated in steady state at a heater temperature of 390 °C. This corresponds to the output results of a total pressure drop of 0.099 MPa in the heat exchangers, maximum operating pressure of 5.7 MPa and 5.8 MPa in the expansion and compression spaces, and total output power of 652 W. In this analysis the optimum operating point of the Stirling engine has been established based only on fixed heater and cooler temperatures of 390 °C and 50 °C. Nevertheless, other input parameters, such as the mean pressure, type of working gas and engine geometry, also determine the most adequate operating mode and conditions; therefore the appropriate definition of these parameters is important for the design of Stirling engines.

For application in the NMT system, the indicated power from the P-V diagram shows that the unit can recover power of 27 Wh/h at a working gas temperature of 390 °C, considering a fuel energy input of 25 kJ/g and faeces generation rate of 200 g/cap/day for a household of ten people [21]. This indicative power is the maximum energy that can be recovered from the Stirling engine at fixed heater and cooler temperatures of 390 °C and 50 °C. In this work, the energy requirements for operating the NMT, such as heat required for drying, igniting and converting the moist faeces, and the energy input into the membrane system, are not considered. However, a previous probabilistic analysis of the power demand and production of the unit has demonstrated that there is high probability that the Stirling engine can supply a power output between 61.5 and 73 W, and that the NMT can achieve a positive net power output between 15.8 and 35 W [30].

### 3.3 Influence of the working gas temperature on the SE power output

A sensitivity analysis was conducted with an assumption of a constant heat loss of 30 °C with an increment of 10 °C in the heat transfer from the flue gas to the working gas via conduction for various working gas temperatures, within the range of 150–390 °C, in order to evaluate the effect on the power output of the Stirling engine. The analysis provided further information on the performance of the engine, and the temperature limit below which the Stirling engine is not efficient. The outcomes of the sensitivity analysis are presented in Table 4. As was expected, the increment in the heat transfer by conduction, the higher the working gas temperature and the output performance of the Stirling engine, expressed in terms of power output and efficiency. Nishiyama et al. [13] presented similar results when evaluating a Stirling engine combined with a simplified biomass combustion in a CHP system. The results obtained in this study indicate the proposed energy recovery system for the NMT unit is thermodynamically feasible considering the output performance of the Stirling engine. With conductive heat transfer from 167 W to 547 W, there was an increment in temperature from 150 °C to 290 °C which gave rise to a 30–50% increase in the power output. While with conductive heat transfer from 601 W to 820 W, a similar increment in temperature from 310 °C to 390 °C resulted in a 10–25% increase in the power output. These results are in good agreement with previous literature on the performance of Stirling engines under various conditions [22], [31], [32], [33]. It is clear that the temperature of the working gas in the Stirling engine is a major determining factor in the output performance of the engine. Therefore, higher operating temperature and optimised design of heat exchangers should be considered in order to obtain high output power and efficiency. A similar approach was discussed by Chmielewski et al. [34].

**Table 4:** Thermodynamic performance of a Stirling engine at different working gas temperatures.

Heat transfer by conduction (W)	Working gas temperature (°C)	Power Output (SE) (Wh/h)	Efficiency (SE) (%)	Pressure drop (MPa)	Heat loss (J)	
164	150	−11.6	−11.45	0.127		16.2
219	170	−7.8	−7.13	0.125		17.7
273	190	−4	−3.43	0.123		18.2
328	210	1.5	1.16	0.122		19.7
383	230	3.2	2.52	0.121		20.2
437	250	6.5	4.99	0.120		21.3
491	270	9.9	7.31	0.119		22.4
547	290	13	9.34	0.119		23.5
601	310	16	11.43	0.118		24.5
656	330	19	13.15	0.114		25.7
711	350	21.8	14.82	0.112		26.9
765	370	24.6	16.57	0.111		28.0
820	390	27.2	17.81	0.103		29.2

As can be seen in Table 4, heat loss increases with the increase in working gas temperature as a result of the increase in heat transfer by conduction. Nevertheless, high heat loss can affect the efficiency of the whole system, and a compromise between the efficiency of the Stirling engine and the overall efficiency needs to be achieved at the experimental stage. Thus, it can also be observed in Table 4, that the pressure drop within the engine decreases as the working gas temperature increases. Leu [35] observed a different behaviour when analysing a Stirling engine integrated directly into an updraft gasifier for power generation with the flue gas produced by the gasifier channelled directly to the heater head of the engine; this author observed that the pressure drop increased to a maximum point and then decreased when increasing the waste heat rate input. The different behaviour is explained based on the different configurations of the gamma Stirling engine, and the accurate calculation of the pressure drops in the heat exchangers carried out in this study, especially in the regenerator, which has a considerable effect on the output performance of the engine considering its effectiveness on the heat transfer to and from the working gas. At the same time, accounting for the pressure drop in the heat exchangers improves the calculation of the heat transfer and the subsequent determination of the thermal efficiency of the system. Precise conclusions on the system performance of the integrated system may not be achieved yet, as the integration concept is still at an early stage, and certain assumptions were made based on the inputs of the Stirling engine, such as the heater and cooler temperature, and those listed in Section 2.4. The numerical analysis will buttress the design parameters that will be utilised in the experimental stage.

### 3.4 Application of the integration of the micro-combustor and Stirling engine in the NMT unit

To achieve higher performance output from the Stirling engine, careful consideration must be given to the air preheating, the design of the heat exchangers, the flue gas temperatures and the heat transfer by conduction from the combustor walls to the Stirling engine in the experimental stage. For optimum performance and for the Stirling engine to generate a power output of 27 Wh/h in the integration with the micro-combustor in the NMT unit, certain factors are required and will be considered in the experimental investigation which is currently underway at Cranfield University.

(1) The finite heat surface area provided by gas flow in the combustor outer wall used as a heat source for the Stirling engine, as the model in this study, considers the constant heat supply from the combustor at a fixed temperature of 390 °C. In order to maximise the power output of the engine, reduce thermal losses and improve conversion efficiency, an additional heat source such as a supplementary burner may be considered.
(2) In this application of heat recovery from the micro-combustor using the Stirling engine, a constant temperature profile is presumed. In the experimental stage, more focus will be directed to the significance of the theoretical and practical implications of waste heat recovered from the hot gas from the micro-combustor, and further analysis will be carried out on the hot waste gas as the heat source for the Stirling engine from the perspective of heat and mass transfer and thermal gradient. Also, alternative devices, such as thermoelectric generators, will be considered and integrated into the NMT system in order to compare the performance on waste heat recovery with that of Stirling engines.
(3) In the practical stage, the velocity of the thermal flow in the micro-combustor will be based on factors such as air-fuel ratio and pressure drop. Coupled with the low viscosity of the gas, the gas phase is inevitably laminar and as such the available heat at the surface (based on contact time and mixing) will reduce the accessibility of all the thermal mass flow. This provides an opportunity, not just about the heat transfer surface area but rather the heat transfer area orientation to manage the heat recovered.

For application in the NMT system, considering a fuel energy input of 25 kJ/g and faeces generation rate of 200 g/cap/day for a household of ten people, the indicated power from the P-V diagram shows that the unit can recover power of 27 Wh/h at a constant working gas temperature of 390 °C from the heat supply via conduction at 820 W from the flue gas. Further work will be carried out on the experimental investigation of the Stirling engine to micro-combustor integration and to achieve a steady state operation of the NMT system as a whole. This will aid the energy recovery process and self-sustainability of the system.

## 4 Conclusions

The main aim of this paper is to analyse the thermodynamic performance of the gamma type Stirling engine integrated into a micro-combustor. The analysis is then related to the case of the NMT unit being designed at Cranfield University.

The temperature of the Stirling engine working gas was defined by an assumption of a constant heat loss of 30 °C with an increment of 10 °C in the heat transfer from the flue gas leaving the micro-combustor to the working gas via conduction. The power output and efficiency of the Stirling engine based on heat transfer by conduction, working gas temperature difference, the thermal losses and pressure drop within the Stirling engine, were investigated in relation to the heat exchangers. At a working gas temperature of 390 °C, the Stirling engine exhibited the highest output power of 27 Wh/h at a thermal efficiency of 17.81%. The overall performance and efficiency of the Stirling engine in relation to the system integration was evaluated and compared with previous results.

It is concluded that the performance of the Stirling engine is very sensitive to the heat transfer by conduction and the temperature of the working gas. The power output of the Stirling increases with an increase in the temperature of the working gas in the case of the integration of the Stirling engine and the NMT unit. This implies that the appropriate location of the Stirling engine regarding the combustor wall is crucial and it has to be taken into account for design purposes.

An average increase of 24% in efficiency of the Stirling engine for every 20 °C increase in temperature of the working gas was observed. The oscillatory flow of the working gas between the heat exchangers and work spaces also increased as a result of the increase in temperature and pressure. The increase in the heat transfer by conduction from the micro-combustor to the Stirling engine and working gas temperature, increased the power output of the Stirling engine and the heat loss. With the regenerator porosity of 0.75, the increase in the working gas temperature decreased and the total pressure dropped in the heat exchangers. Hence, careful consideration should be given to the regenerator design due to the hydraulic resistance when the working gas flows through it, as this can strongly affect the power output of the Stirling engine.

The concept results presented in this paper point out that, in the case of an actual integration of the micro-combustor and Stirling engine at an experimental stage, the heat input and heat transfer rate from the flue gas to the working gas of the Stirling engine have to be thoroughly considered and their effect on the output performance of the Stirling engine and overall efficiency of the NMT unit has to be investigated.
